# The Interplay Between Water Limitation, Dhurrin, and Nitrate in the Low-Cyanogenic Sorghum Mutant *adult cyanide deficient class 1*

**DOI:** 10.3389/fpls.2019.01458

**Published:** 2019-11-15

**Authors:** Viviana C. Rosati, Cecilia K. Blomstedt, Birger Lindberg Møller, Trevor Garnett, Ros Gleadow

**Affiliations:** ^1^School of Biological Sciences Faculty of Science Monash University, Clayton, Victoria, Australia; ^2^Plant Biochemistry Laboratory and VILLUM Research Centre for Plant Plasticity, Department of Plant and Environmental Sciences, University of Copenhagen, Copenhagen, Denmark; ^3^The Australian Plant Phenomics Facility, The University of Adelaide, Adelaide, Australia

**Keywords:** cyanogenesis, cyanogenic glucosides, drought, resource allocation, specialized metabolites

## Abstract

*Sorghum bicolor* (L.) Moench produces the nitrogen-containing natural product dhurrin that provides chemical defense against herbivores and pathogens via the release of toxic hydrogen cyanide gas. Drought can increase dhurrin in shoot tissues to concentrations toxic to livestock. As dhurrin is also a remobilizable store of reduced nitrogen and plays a role in stress mitigation, reductions in dhurrin may come at a cost to plant growth and stress tolerance. Here, we investigated the response to an extended period of water limitation in a unique EMS-mutant *adult cyanide deficient class 1* (*acdc1*) that has a low dhurrin content in the leaves of mature plants. A mutant sibling line was included to assess the impact of unknown background mutations. Plants were grown under three watering regimes using a gravimetric platform, with growth parameters and dhurrin and nitrate concentrations assessed over four successive harvests. Tissue type was an important determinant of dhurrin and nitrate concentrations, with the response to water limitation differing between above and below ground tissues. Water limitation increased dhurrin concentration in the *acdc1* shoots to the same extent as in wild-type plants and no growth advantage or disadvantage between the lines was observed. Lower dhurrin concentrations in the *acdc1* leaf tissue when fully watered correlated with an increase in nitrate content in the shoot and roots of the mutant. In targeted breeding efforts to down-regulate dhurrin concentration, parallel effects on the level of stored nitrates should be considered in all vegetative tissues of this important forage crop to avoid potential toxic effects.

## Introduction

Cyanogenic glucosides are specialized secondary metabolites, produced by over 2,500 species of plants and found in one-third of crop species (Gleadow and Møller, 2014). The role of cyanogenic glucosides in plant defense has long been established (Jones, 1998; Gleadow and Woodrow, 2002a; Zagrobelny et al., 2004), with defense theories assuming their production comes at a direct cost to primary metabolism when resources are limited (Herms and Mattson, 1992; Neilson et al., 2013; Cipollini et al., 2014,).

The line between primary and secondary metabolism becomes blurred as cyanogenic glucosides are a remobilizable store of reduced nitrogen, transport compounds, and enhancers of stress tolerance as they mitigate oxidative stress (Møller, 2010; Burke et al., 2013; Pičmanová et al., 2015; Gleadow et al., 2016b; Bjarnholt et al., 2018; Schmidt et al., 2018). In stressed plants, where photosynthetic rate is reduced, cyanogenic glucosides may also provide a ready source of nitrogen, remobilized when the stress is alleviated (Selmar et al., 1988; Kongsawadworakul et al., 2009; O’Donnell et al., 2013; Bjarnholt et al., 2018; Schmidt et al., 2018). The cross-over of cyanogenic glucosides for use in primary and secondary metabolism is demonstrated by the negative effects on plant growth at specific developmental stages when they are reduced or removed, as seen in cassava (*Manihot esculenta* Crantz) (Jørgensen et al., 2005) and the acyanogenic sorghum line *totally cyanide deficient 1* (*tcd1*) (Blomstedt et al., 2012; Blomstedt et al., 2018).

Sorghum [*Sorghum bicolor* (L.) Moench] contains the cyanogenic glucoside dhurrin [(*S*)-4-hydroxymandelonitrile-β-*D*-glucopyranoside] in all main tissues except the mature grain (Kahn et al., 1997; Bak et al., 1998; Nielsen et al., 2016). Following tissue disruption, for example as a result of herbivore feeding, dhurrin is brought into contact with the endogenous β-glucosidase dhurrinase, resulting in hydrolysis of the glucoside and the release of hydrogen cyanide gas (HCN), also known as prussic acid (Kahn et al., 1997; Cicek and Esen, 1998). Dhurrin content varies with the ontogeny of the sorghum plant, increasing rapidly post-germination where it can reach up to 30% dry mass of the shoot tip before decreasing as the plant matures (Halkier and Møller, 1989; Adewusi, 1990; Busk and Møller, 2002). New growth also has high dhurrin concentrations presumably to ensure that such tissues, which are particularly vulnerable to herbivory, are chemically defended (Gleadow and Woodrow, 2002a).

Developmental regulation of dhurrin formation in sorghum is confounded by environmental factors such as drought and nitrogen application that can induce higher dhurrin concentrations (O’Donnell et al., 2013; Neilson et al., 2015; Gleadow et al., 2016a; Blomstedt et al., 2018; Emendack et al., 2018). This renders crop toxicity difficult to predict. Toxicity predictions are further complicated due to high variability between lines and individuals within lines (Hayes et al., 2015; Emendack et al., 2018). Moreover, the degree of HCN induction appears to differ depending on whether the stress is chronic or acute (Wheeler et al., 1990).

Sorghum also accumulates nitrate, particularly in the sheath tissue (O’Donnell et al., 2013; Blomstedt et al., 2018). Like dhurrin, nitrate concentrations are affected by the environment. Drought stress adds to the accumulation of nitrate as the ability to assimilate nitrate into protein is reduced (Sivaramakrishnan et al., 1988). There is conflicting evidence as to whether lower dhurrin concentrations are associated with higher nitrate levels in sorghum (O’Donnell et al., 2013; Neilson et al., 2015; Gleadow et al., 2016a; Blomstedt et al., 2018). Furthermore, whether there is a stoichiometric trade-off in the allocation of nitrogen to dhurrin and nitrate remains unclear.

In this study, we used a sorghum mutant line with altered cyanogenic potential to investigate the effect of an extended period of water limitation on dhurrin and nitrate concentrations, as well as the allocation of nitrogen to both compounds. The *adult cyanide deficient class 1* (*acdc1*) is a sorghum EMS-mutant identified from a TILLING population (Blomstedt et al., 2012). This developmental mutant has wild-type concentrations of dhurrin in all vegetative tissues when the plants are young. When reaching 3 to 4 weeks of age, the *acdc1* mutant decreases the dhurrin concentration in its leaf tissue more rapidly than wild-type plants (Blomstedt et al., 2012). By 5 weeks of age, the *acdc1* mutant has significantly lower dhurrin concentrations in the leaf tissue in comparison to wild-type plants, and by 8 weeks of age, only negligible concentrations of dhurrin remain in the *acdc1* leaf tissue (Blomstedt et al., 2018). No sequence changes in the coding regions of the dhurrin biosynthetic genes (*CYP79A1*, *CYP71E1* and *UGT85B1*) are present in *acdc1*. Complementation tests suggest the *acdc1* causal mutation is in the *CYP79A1* promoter region and a CΔT substitution (consistent with EMS treatment) that segregates with the phenotype has been identified ∼1.1 kb upstream of the *CYP79A1* transcription start site (Rosati et al., 2019). The mutation is recessive, with individuals requiring two copies of the CΔT mutation to display the *acdc1* phenotype, while heterozygotes display a wild-type phenotype throughout development (Rosati et al., 2019). Sibling lines (Sibs) that lack the *acdc1* mutation were generated in parallel to account for any effect of background mutations generated from the EMS treatment. The *acdc1* and Sibs were backcrossed three times to wild-type sorghum to reduce background mutations and appear phenotypically normal except for the altered cyanogenic status present in *acdc1*.

Limiting water in greenhouse experiments in a manner that is both precise and equivalent to the chronic stress sorghum can experience in the field is known to be difficult (Flower et al., 1990; Tangpremsri et al., 1991; Passioura, 2006; Sabadin et al., 2012). In this study, we used a gravimetric platform allowing for precise and reproducible water application, ensuring low soil water levels were maintained in a highly accurate manner throughout the course of the experiment. The platform allows the exact volume of water applied to each individual plant to be monitored. Plants were grown at either 15%, 30%, or 100% field capacity of water. As sorghum is a highly drought-tolerant C_4_ crop with an extensive root system and thick, waxy cuticle on the leaves, the 15% and 30% field capacity of water treatments were selected to elicit a chronic stress response. The experiment was undertaken over a 35-day period, with a baseline harvest at 11 days post-germination (dpg) followed by three destructive harvests occurring every 8 days (19, 27, and 35 dpg).

In this study we investigated the effects of an extended period of water limitation on a sorghum mutant line with altered cyanogenic potential in comparison to wild-type plants. This enabled us to assess how an altered hydrogen cyanide potential coupled with water limitation affects nitrate concentrations and nitrogen allocation in both above and below ground tissues. Successive harvests during early development also enabled the interplay between developmental and environmental regulation of dhurrin to be investigated.

## Methods

### Plant Material and Growth Conditions

Seeds from the wild-type line BTx623, mutant line *adult cyanide deficient class 1* (*acdc1*), and mutant sibling line (Sibs) (Blomstedt et al., 2012) were used to analyze the effects of water limitation on growth, hydrogen cyanide potential (HCNp), and nitrate concentrations. The plants were grown using an automated gravimetric platform watering system (Phenospex Droughtspotter, Heerlen, The Netherlands) at the Australian Plant Phenomics Facility, Adelaide, South Australia, during January–February 2016. The system consists of 168 individual lysimeters, each connected to a separate watering spigot that applies water to the top of a pot. Pots are arranged in two 3 × 28 grids in a spilt–split plot design (Cousins et al., 2019). Individual pots were weighed every 10 min, allowing for water use in each pot to be quantified, with water added when pots were 0.5% below target weight. Six pots were used as plant-free controls to allow for the calculation of evaporative water loss. The plants received natural light with the daily light integral (DLI) over the duration of the experiment being 10 mol m^-2^ day^-1^. Temperatures ranged from 16°C at night to 26°C during the day with a daily average of 22°C. Relative humidity was 80% at night and 50% during the day.

Three seeds of each line were germinated in 20-cm free-draining pots containing 4.5 L of soil, 50% (v/v) University of California (UC) mixture (1:1 peat:sand), and 50% (v/v) cocopeat amended with Osmocote. At the 2-leaf stage, plants were thinned to one per pot with a focus on overall plant uniformity. A baseline harvest of 6 plants from each line was undertaken at 11 days post-germination (dpg) before treatments commenced. Following this, the gravimetric platform was used to establish three watering regimes. These are referred to as 100% field capacity of water (near saturation and appropriate for the support of unimpeded sorghum growth), 30% field capacity of water, and 15% field capacity of water. Field capacity of the soil mixture was established following the protocol of Cousins et al. (2019). Plants were destructively harvested every 8 days for three additional harvests, resulting in harvests at 19, 27, and 35 dpg. Fifty-four plants were harvested at each harvest interval; six from each of the three lines (wild type, Sibs, *acdc1*) for each watering regime (100%, 30%, and 15% field capacity of water). Harvest intervals were selected to cover the commencement of the ontogenic reduction in HCNp, seen in the *acdc1* (Blomstedt et al., 2012).

At each harvest plant tissues were separated into leaf blades (removed at the ligule), sheaths (comprising the rolled leaf sheaths and the shoot stem), and roots which were brushed free of soil, except for the 11 dpg harvest where leaf and sheath tissues were harvested together due to the small size of the plants. Division of tissues allowed for chemical analyses to be undertaken for each tissue type. Fresh mass of each tissue was recorded, and leaf blade area was measured using the LI-COR 3000 leaf area meter (LI-COR Lincoln, Nebraska, USA). Leaf, sheath, and root tissues were snap frozen in liquid nitrogen and stored at −80°C until freeze dried. Freeze-dried tissue was weighed and ground to a fine powder using a MixerMill (MM 300, Retsch, Hann, Germany).

### Growth Indices

Dry matter percentage (DM%), relative growth rate (RGR), net assimilation rate (NAR), leaf area ratio (LAR), specific leaf area (SLA), and specific leaf nitrogen (SLN) were derived from the harvest data using the following equations, after Gleadow and Rowan (1982):

$$DM\%=\left(\frac{DW}{FW}\right)100$$

$$RGR\;d^{-1}=\frac{lnW_{2}-lnW_{1}}{t_{2}-t_{1}}$$

$$NAR\;(g\;m^{-2}\;d^{-1})=\left(\frac{W_{2}-W_{1}}{t_{2}-t_{1}}\right)\left(\frac{lnA_{2}-lnA_{1}}{t_{2}-t_{1}}\right)$$

$$LAR\;(m^{2}\;g^{-1})=\;\frac{A}{W\;total\;}$$

$$SLA\;(m^{2}g^{-1})=\;\frac{A}{W\;leaf}$$

$$SLN\;g\;g^{-1}=(leaf\;N)\left(\frac{W_{L}}{A_{L}}\right)$$

where DW is dry weight; FW is fresh weight; W is total biomass; W_L_ is leaf biomass; A is leaf area; t is time; and N is leaf nitrogen.

### Chemical Analyses

Hydrogen cyanide potential (HCNp) was determined at all harvest time points using 10 mg of finely ground leaf, sheath, or root tissue. HCNp is the total amount of HCN produced by hydrolysis of the entire content of endogenous cyanogenic glucosides that is achieved by adding exogenous β-glucosidase (β-D-Glucoside glucohydrolase, G4511, Sigma-Aldrich, Sydney, Australia). The HCN produced was captured as NaCN in a 1M NaOH solution and measured via a colorimetric assay (Gleadow et al., 2012). The HCNp is used as a proxy for dhurrin, with each milligram of HCN equivalent to 11.5 mg of dhurrin in the plant tissue. Total nitrate concentration was measured for the final harvest time point via a colorimetric assay in 96-well microtiter plates using 15 mg of finely ground freeze-dried leaf, sheath, or root tissue (O’Donnell et al., 2013). Total nitrogen of the leaf, sheath, and root samples for the final harvest was analyzed using 5 mg of tissue by the Environmental Analysis Laboratory (Lismore, NSW, Australia). Nitrogen, dhurrin, and nitrate per plant was converted to mg g^-1^ dry mass. In order to assess how nitrogen was partitioned, the proportion of nitrogen found as dhurrin or nitrate was calculated as a proportion of total elemental nitrogen.

### Statistical Analysis

For the baseline harvest at 11 dpg, plants of each line were randomly selected during the thinning process with data analyzed by one-way ANOVA. For the harvests at 19, 27, and 35 dpg, a split–split plot design was used to assign the three treatments and three harvest times to the 54 plants of each line. Water levels were assigned to whole plots using a randomized complete block design with three replicates. Each whole plot was split into three subplots to which there were three randomized harvest times; and each subplot was split into three, to which genotypes were randomized. As plants were destructively harvested at each time point, this was not a repeated measurements experiment. Each harvest was analyzed separately using a split-plot ANOVA performed with Minitab 19 (Minitab Software, State College, PA). For all tests, a *P*-value of <0.05 was considered significant. Means that were significantly different were compared *post hoc* using Tukey’s test. If required, data were log-transformed in order to satisfy the assumptions of normality.

## Results

### Physiology and Growth

EMS mutants with low adult dhurrin levels (*adult cyanide deficient class 1*, *acdc1*), mutant sibling lines (Sibs, generated in parallel but lacking the mutation), and wild-type (WT) plants were grown for 5 weeks under well-watered or water-limited conditions (15%, 30%, and 100% field-capacity of water).

**Figure 1:**
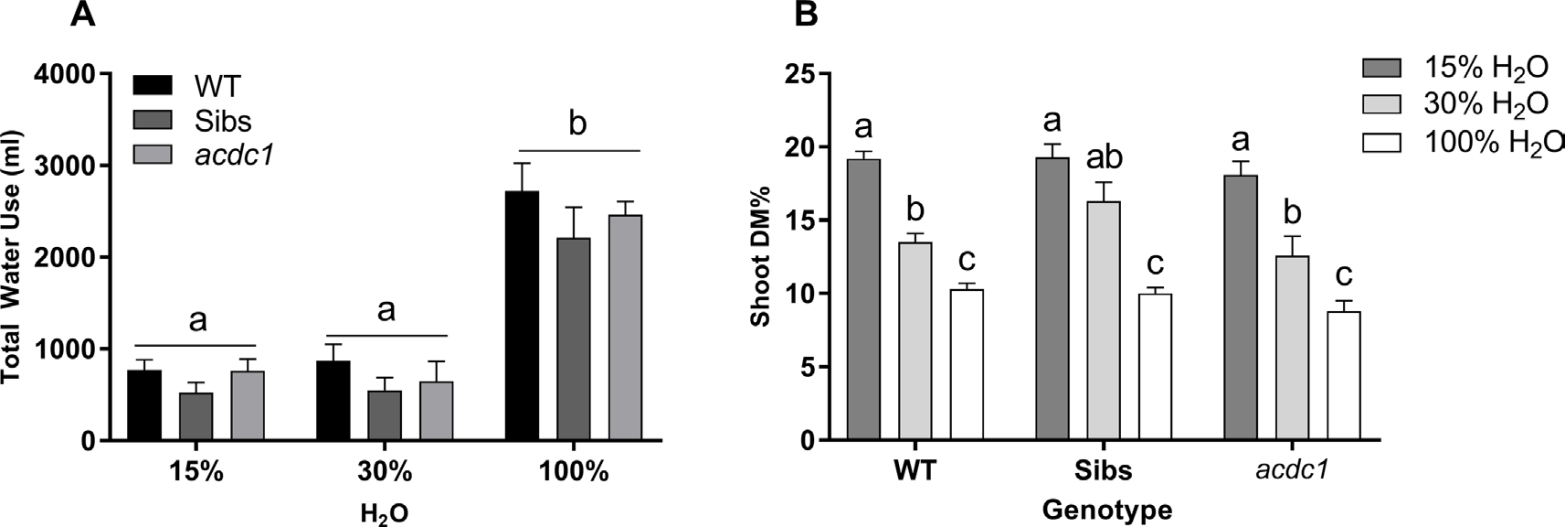
**(A)** Total water use and **(B)** shoot dry matter percentage (DM%) of wild-type: WT; Siblings: Sibs; and *adult cyanide deficient class 1*: *acdc1* lines grown at 15%, 30%, and 100% field capacity of water. Shoot dry matter percentage increased as water availability decreased, demonstrating that all lines were significantly affected by both levels of water limitation. Values denote mean ± 1SE (*n*=3); means with different letters are significantly different at P < 0.05 analyzed using ANOVA and Tukey’s test. Data represent plants at 35 days post-germination (dpg); treatments commenced at 11 dpg.

Water availability was the greatest determinant of plant growth at all harvest time points for all lines. These differences were not proportional to water availability, with few significant differences seen between the 15% H_2_O and 30% H_2_O treatments. This was congruent with the amount of water used over the course of the experiment, with no significant differences in total water use between the 15% H_2_O and 30% H_2_O treatments, while the 100% H_2_O treatment resulted in an almost tripled amount of water consumed (P < 0.01) (**Figure 1A**). Dry-matter percentage (DM%) correlated to water restriction levels, increasing as water availability decreased (**Figure 1B**). No differences between lines in relation to DM% were observed.

The first harvest showed few genotype effects, with the only significant difference between lines seen in the *acdc1*, which had a greater specific leaf area (SLA) than the two other lines (P < 0.001) (**Supplementary Table 1A**). Once treatments commenced, the *acdc1* line had significantly greater root mass (P < 0.05) than wild-type plants at 100% H_2_O (**Supplementary Table 1B**). The main genotype effect occurred in the Sibs, which by 35 dpg had reduced height, leaf area, leaf mass, and sheath mass at 15% H_2_O and 30% H_2_O than both the wild-type and *acdc1* lines (P < 0.001) (**Table 1**).

**Table 1:** Growth parameters of three *Sorghum bicolor* lines grown at 15%, 30%, and 100% field capacity of water at 35 days post germination.

	WT			Sibs			*acdc1*			ANOVA		
	15% H_2_O	30% H_2_O	100% H_2_O	15% H_2_O	30% H_2_O	100% H_2_O	15% H_2_O	30% H_2_O	100% H_2_O	L	T	LxT
Leaf area (cm^2^)	110 (10)	280 (40)	880 (110)	60 (10)	120 (20)	660 (60)	160 (30)	350 (50)	1050 (130)	*	***	ns
Height (cm)	10.5 (0.8)	14.7 (1.1)	23.0 (0.4)	7.4 (0.4)	7.9 (0.8)	18.0 (1.7)	8.5 (0.7)	13.2 (1.1)	22.8 (0.9)	*	**	ns
Leaf DM (g)	0.34 (0.05)	0.68 (0.12)	2.42 (0.41)	0.18 (0.02)	0.28 (0.04)	1.87 (0.25)	0.45 (0.09)	0.83 (0.20)	2.92 (0.80)	*	***	ns
Sheath DM (g)	0.19 (0.03)	0.32 (0.06)	1.28 (0.22)	0.09 (0.01)	0.13 (0.02)	0.90 (0.14)	0.45 (0.24)	0.42 (0.08)	1.50 (0.25)	*	**	ns
Root DM (g)	0.67 (0.12)	1.21 (0.27)	4.68 (1.75)	0.56 (0.13)	0.88 (0.24)	2.11 (0.46)	0.45 (0.08)	1.24 (0.47)	3.83 (1.18)	ns	**	ns
Biomass total (g)	1.19 (0.15)	2.21 (0.43)	8.37 (2.37)	0.83 (0.15)	1.31 (0.30)	4.88 (0.81)	1.35 (0.31)	2.49 (0.71)	8.25 (2.09)	*	***	ns
R:S	1.4 (0.3)	1.2 (0.1)	1.1 (0.3)	2.1 (0.4)	2.0 (0.5)	0.7 (0.1)	0.6 (0.1)	0.9 (0.2)	0.9 (0.2)	ns	ns	ns
LAR (m^2^ g^-1^)	100 (10)	130 (10)	130 (20)	80 (10)	100 (30)	140 (10)	140 (20)	160 (20)	150 (20)	ns	ns	ns
SLA (m^2^ g^-1^)	350 (20)	430 (20)	380 (30)	350 (20)	410 (30)	360 (20)	370 (30)	490 (100)	410 (70)	ns	*	ns
SLN (g g^-1^)	0.4 (0.1)	0.7 (0.20)	3.2 (1.0)	0.2 (0.02)	0.3 (0.04)	2.2 (0.4)	0.6 (0.1)	0.9 (0.3)	3.9 (1.3)	ns	**	ns
NAR (g m^-2^ day^-1^)	0.01 (0.002)	0.03 (0.01)	0.1 (0.06)	0.01 (0.002)	0.02 (0.006)	0.05 (0.02)	0.02 (0.01)	0.05 (0.020)	0.09 (0.03)	ns	ns	ns
RGR (g g^-1^ day^-1^)	0.15 (0.004)	0.17 (0.01)	0.22 (0.01)	0.13 (0.01)	0.15 (0.01)	0.21 (0.01)	0.15 (0.01)	0.18 (0.01)	0.23 (0.02)	*	**	ns

WT, wild type; Sibs, siblings; acdc1, adult cyanide deficient class 1 mutants. Values denote mean ± 1SE (n=3). Means with different letters are significantly different at P < 0.05 analyzed using ANOVA and Tukey’s test. DM, Dry mass; LAR, leaf area ratio; NAR, net assimilation rate; R:S ratio, root/shoot ratio; SLA, specific leaf area; SLN, specific leaf nitrogen; RGR, relative growth rate. L,Line; T,Treatment; *P < 0.05; **P < 0.01; ***P < 0.001; ns, not significant. Growth parameters for earlier harvests (11, 19, and 27 dpg) are detailed in **Supplementary Table 1**.

Water availability began to affect plant growth at 19 dpg, when treatments had been applied for 8 days. Height, leaf area, and leaf dry mass were lower in the 15% H_2_O and 30% H_2_O treatment groups in comparison to fully watered plants across all genotypes (P < 0.05) (**Supplementary Table 1B**), but no significant differences between the 15% H_2_O and 30% H_2_O treatments were observed. Both levels of water restriction resulted in an equivalent growth delay.

Height, leaf area, leaf dry mass, and sheath dry mass remained lower at 15% H_2_O and 30% H_2_O in comparison to fully watered plants for the final two harvests (P < 0.001) (27 dpg—**Supplementary Table 1C**; and 35 dpg—**Table 1**). This corresponded to a greater total biomass in the fully watered plants (P < 0.01). No significant difference in total biomass was observed between the 15% H_2_O and 30% H_2_O treatments within lines. Higher SLN and RGR was seen at the final harvest (35 dpg) at 100% H_2_O in comparison to both levels of water limitation for all genotypes (P < 0.01) (**Table 1**).

### Plant Composition: HCNp, Nitrate Concentration, and Total N of Well-Watered and Water-Limited Plants

#### HCNp Analysis

**Figure 2:**
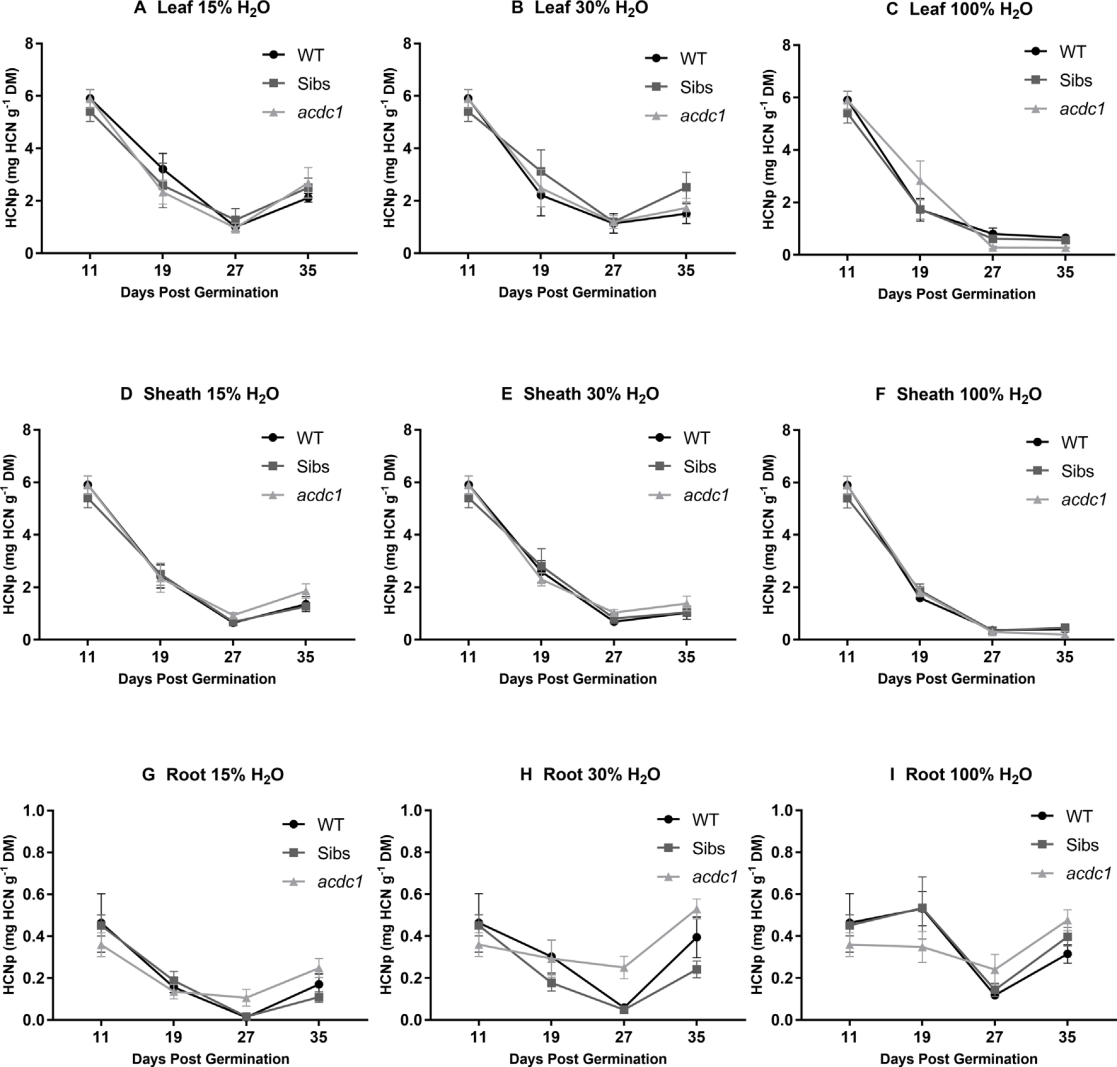
Hydrogen cyanide potential (HCNp; mg HCN g^-1^ dry mass) in the leaves **(A**–**C)**; sheaths **(D**–**F)**; and roots **(G**–**I)** of WT, wild-type; Sibs, siblings; and *acdc1*: *adult cyanide deficient class 1* sorghum lines grown at 15%, 30%, and 100% field capacity of water. A baseline harvest (prior to water limitation) occurred at 11 days post-germination (dpg), followed by harvests at 19, 27, and 35 dpg. Values denote mean ± 1SE (*n*=3); significance is listed in **Supplementary Table 2**, analyzed using ANOVA and Tukey’s test.

Hydrogen cyanide potential (HCNp; mg HCN g^-1^ DM) was analyzed in three tissue types for each line at each harvest (**Figure 2**, **Supplementary Table 2**). HCNp was highly dependent on developmental stage and tissue type, with water availability not resulting in significant differences in HCNp until the later harvests (**Figure 2**). HCNp decreased markedly in both the leaf (**Figures 2A–C**) and sheath tissue (**Figures 2D–F**) between 11 and 27 dpg in all lines and treatments. This decrease was more pronounced at 100% H_2_O and by 27 dpg HCNp in the shoot was 0.46 ± 0.08 mg HCN g^-1^ DM on average for all lines, compared with 0.92 ± 0.19 and 1.01 ± 0.19 mg HCN g^-1^ DM in the 15% H_2_O and 30% H_2_O treatments, respectively. Root tissue had approximately 80% lower HCNp than the shoot tissue at the baseline harvest (**Figures 2G–I**). HCNp in the root tissue displayed a similar pattern to the shoot tissue, with a general decrease in HCNp occurring from 19 to 27 dpg (except in the *acdc1*), before an increase across all treatments at 35 dpg (**Figures 2G–I**; **Supplementary Table 2**).

There were genotype differences in HCNp of the leaves and roots in plants grown at 100% H_2_O. For example, in the leaves *acdc1* had significantly lower HCNp (0.29 ± 0.05 mg HCN g^-1^ DM) at 27 dpg than the wild type (0.78 ± 0.27 mg HCN g^-1^ DM) or Sibs (0.67 ± 0.06 mg HCN g^-1^ DM) (**Figure 2**; P < 0.05). The *acdc1* leaf tissue continued to have significantly lower HCNp through to the final harvest compared to both the other lines, with an average HCNp of 0.28 ± 0.02 mg HCN g^-1^ DM compared with 0.65 ± 0.06 and 0.60 ± 0.03 mg HCN g^-1^ DM for the wild-type and Sibs lines, respectively. There was no significant difference in sheath HCNp between lines.

In the roots, genotype differences were observed at 27 dpg. In contrast to the leaf tissue, *acdc1* had a HCNp at 100% H_2_O of 0.24 ± 0.03 mg HCN g^-1^ DM, approximately twice as high as HCNp in the roots of wild-type plants (0.12 ± 0.02 mg HCN g^-1^ DM) (**Figure 2**, **Supplementary Table 2**). At 35 dpg, *acdc1* roots still had a significantly higher HCNp, although the difference was not as great as at the earlier harvests.

Water limitation affected the HCNp differently in the above and below ground tissues (**Figure 2**, **Supplementary Table 2**). Leaf and sheath HCNp was significantly higher in plants grown at 15% H_2_O and 30% H_2_O compared to plants grown at 100% H_2_O across all genotypes (P<0.05) (**Supplementary Tables 2A, B**). For example, at 35 dpg average leaf HCNp for the three lines was only 0.51 ± 0.04 mg HCN g^-1^ DM at 100% H_2_O compared to 2.43 ± 0.38 mg HCN g^-1^ DM and 1.92 ± 0.43 mg HCN g^-1^ DM in plants grown at 15% H_2_O and 30% H_2_O, respectively. In contrast to the shoot tissue, the roots had significantly higher HCNp at 100% H_2_O compared to plants grown at 15% H_2_O for all genotypes from the 27 dpg harvest onwards (**Supplementary Table 2C**).

#### Nitrate Concentration

**Figure 3:**
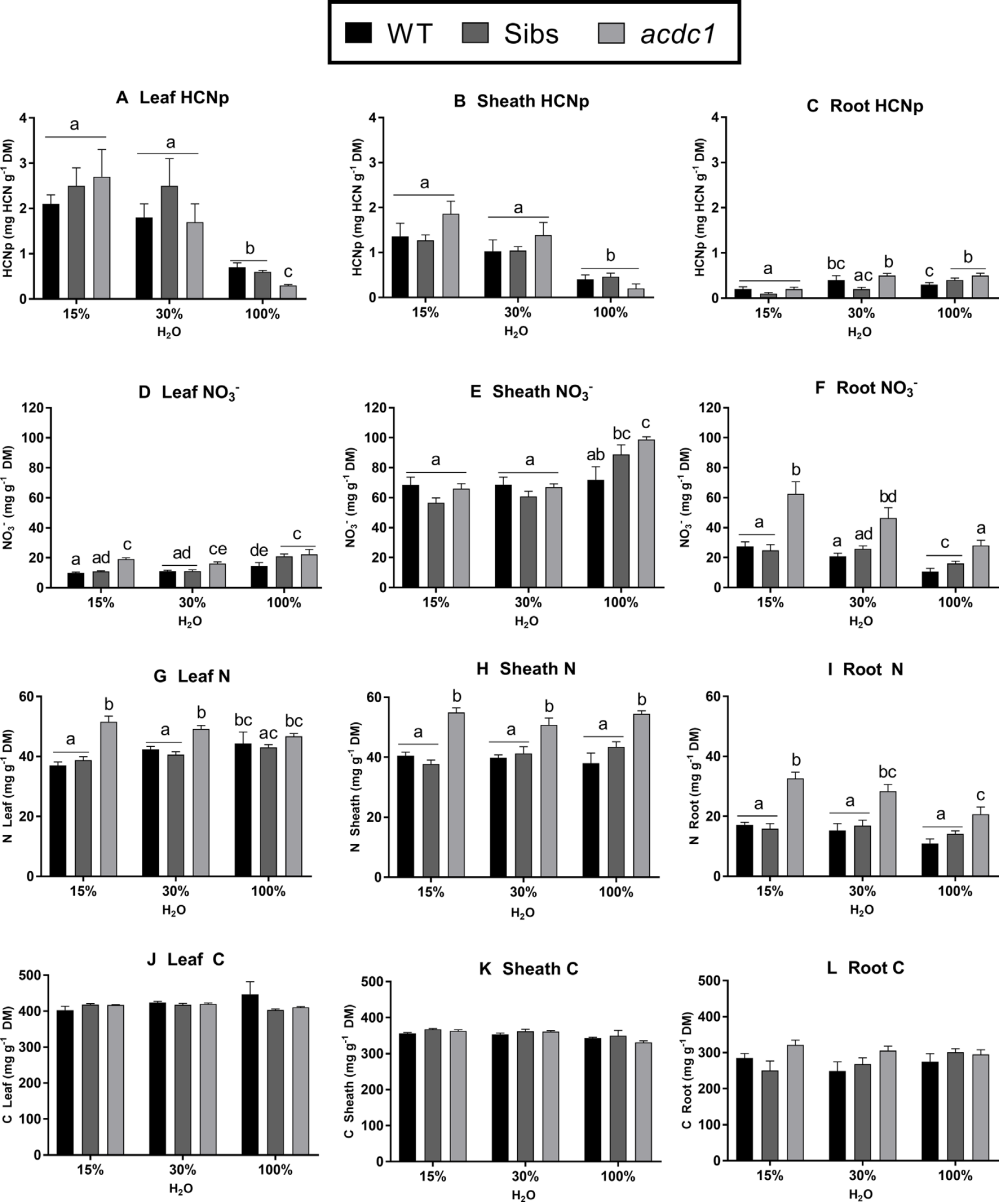
Hydrogen cyanide potential (HCNp; mg HCN g^-1^ dry mass) **(A**–**C)**; nitrate concentration **(D**–**F)**; nitrogen **(G**–**I)**; and carbon **(J**–**L)** in the leaves, sheaths, and roots of WT, wild-type; Sibs: siblings; and *acdc1*, *adult cyanide deficient class 1* sorghum lines grown at 15%, 30%, and 100% field capacity of water at 35 days post-germination. Values denote mean ± 1SE (*n*=3); means with different letters are significantly different at P < 0.05 analyzed using ANOVA and Tukey’s test.

Genotype, tissue type, and water availability all affected nitrate concentration. Leaf and root nitrate concentrations were higher in *acdc1* than both other lines when water was limited (**Figures 3D–F**). In the *acdc1*, nitrate concentrations were also higher than wild-type plants in all tissues at 100% H_2_O (P < 0.01) (**Figures 3D–F**) and higher in the roots than wild-type plants for all treatments (P < 0.01) (**Figure 3F**). Nitrate concentrations were tissue-dependent across all lines, with nitrates highly concentrated in the sheath tissue compared to the leaf tissue (**Figures 3D, E**). When water was limited, root nitrate concentration increased in all lines (**Figure 3F**).

#### Total Nitrogen Concentration

Total elemental N concentration in each tissue followed similar patterns to nitrate concentration (**Figures 3G–I**). The *acdc1* leaves had higher N concentrations than the wild type and Sibs under both levels of water limitation, but not in the well-watered plants (P < 0.01) (**Figure 3G**). In the sheath and root tissues, *acdc1* had higher N concentrations than the wild-type and Sibs for all treatments. (P < 0.001) (**Figures 3H, I**). Nitrogen concentration was lower in the roots in comparison to leaf and sheath tissues, which were comparable. No significant differences were observed in carbon concentration between treatment groups or genotypes (**Figures 3J–L**).

### Nitrogen Allocation

**Figure 4:**
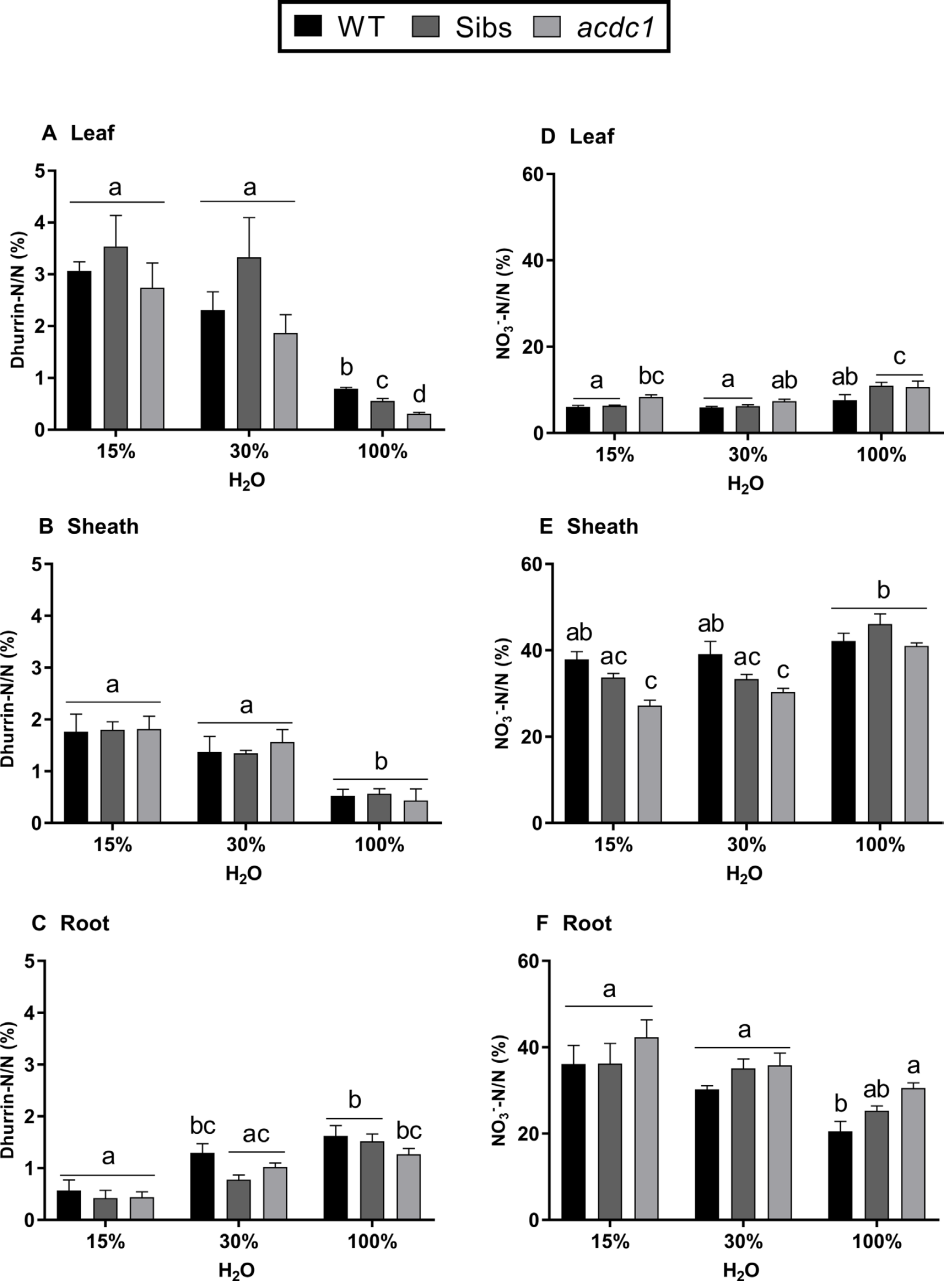
Proportion of nitrogen allocated to dhurrin **(A**–**C)**; and nitrate **(D**–**F)** in the leaves, sheaths, and roots of WT, wild-type; Sibs, siblings; and *acdc1*: *adult cyanide deficient class 1* sorghum lines grown at 15%, 30%, and 100% field capacity of water at 35 days post-germination. Values denote mean ± 1SE (*n*=3); means with different letters are significantly different at P < 0.05 analyzed using ANOVA and Tukey’s test.

In order to analyze nitrogen allocation, we calculated the total amount of dhurrin and nitrate per plant (by multiplying the concentration in each tissue by the biomass of that tissue) before expression as a percentage of total N. When water was replete, wild type and Sibs allocated more N to dhurrin in the leaf compared to *acdc1* (P < 0.05) (**Figure 4A**). This genotypic difference was not seen when water was limited, with more N allocated to dhurrin in both leaf and sheath tissues compared to the 100% H_2_O treatment across all lines (**Figures 4A, B**). Root tissue displayed an opposite pattern to the shoots with more N allocated to dhurrin under fully watered conditions in comparison to 15% H_2_O for all lines (P < 0.01) (**Figure 4C**).

The proportion of nitrogen found in nitrate was tissue-dependent more than water-dependent, with up to six times more N allocated to nitrate in the sheath and root tissues than the leaf tissue across all lines (**Figures 4D–F**). There were also significant genotype effects. For example, the proportion of N allocated to nitrate was significantly higher in *acdc1* leaves than the wild-type when water was both replete and severely limited (15% H_2_O) (P < 0.05) (**Figure 4D**). Less N was allocated to nitrate in the *acdc1* sheath than in the wild-type sheath at both 15% H_2_O and 30% H_2_O (P < 0.01), possibly due to the higher percentage of N in the *acdc1* sheath. In the root tissue, more N was allocated to nitrate in the *acdc1* roots than the wild-type line at 100% H_2_O (P < 0.05) (**Figure 4F**).

## Discussion

Drought is known to affect concentrations of cyanogenic glucosides in many species, generally affecting an increase in concentration in both field and controlled environments as reported in cassava (Brown et al., 2016), eucalypt (*Eucalyptus cladocalyx*) (Gleadow and Woodrow, 2002b), white clover (*Trifolium repens*) (Hayden and Parker, 2002), and lima bean (*Phaseolus lunatus*) (Ballhorn et al., 2011). In sorghum, the concentration of dhurrin may increase or decrease depending on tissue-type and the length and severity of stress (Wheeler et al., 1990; O’Donnell et al., 2013; Gleadow et al., 2016a; Emendack et al., 2018). Increases in dhurrin appear to be associated with chronic stress (Wheeler et al., 1990), though reproducing precise levels of water limitation over extended periods and under controlled conditions is challenging (Flower et al., 1990; Tangpremsri et al., 1991; Passioura, 2006; Sabadin et al., 2012). In the past, experiments of this nature were executed by manual watering to weight, which, due to logistical constraints, cannot be done regularly enough and leads to considerable variation in water stress. In our current study, a gravimetric platform was used, enabling low levels of water to be accurately maintained over the course of the experiment.

Cyanogenic glucosides, in addition to their known role in herbivore defense (Gleadow and Woodrow, 2002a), may help mitigate drought stress such that less cyanogenic plants would have reduced growth during extended periods of water limitation than those with higher concentrations (Gleadow and Møller, 2014). We compared the growth and chemical composition at three different levels of water using the publicly available sorghum breeding line BTx623 and a mutant line *acdc1* with low dhurrin concentration in adult leaf tissue (Blomstedt et al., 2012) grown at three different levels of water. As cyanogenic glucosides also play important roles in nitrogen metabolism, resulting in an interplay between HCNp and nitrate (Selmar et al., 1988; Gleadow and Møller, 2014; Bjarnholt et al., 2018; Blomstedt et al., 2018), we also determined nitrate concentration and nitrogen allocation in the different genotypes.

Overall, the *acdc1* sorghum mutant did not display a growth advantage or disadvantage when water was limited in comparison to wild-type sorghum plants. Plants grown at 15% and 30% field capacity of water showed an equivalent reduction in water use (**Figure 1A**), leading to a reduction in biomass and an overall increase in shoot dry-matter content proportional to the level of water limitation in all lines tested (**Figure 1B**). Both levels of water limitation reduced biomass to the same degree across lines (**Table 1**). The overall relative growth rate (RGR) followed the same pattern, with slower plant growth when water was limited, but with no significant differences in RGR seen between the 15% H_2_O and 30% H_2_O treatments (**Table 1**). It would be interesting to determine whether or not such changes in nitrogen allocation ultimately affect grain yield.

### Water Limitation Overrides the Developmental Decrease of Dhurrin in *acdc1*

We observed that HCNp during early growth was predominantly dependent on developmental stage and tissue type, while the effects of water availability and genotype became significant at the later harvests. These findings are consistent with other studies (Vanderlip, 1972; Miller et al., 2014; Gleadow et al., 2016a). Developmental regulation of dhurrin content, which causes a rapid decrease in HCNp from 4dpg onwards (Halkier and Møller, 1989; Busk and Møller, 2002), was the driving factor of HCNp during the early stages of plant growth, with a decrease in dhurrin concentration in the leaf and sheath tissue from 11 to 27 dpg observed across all lines and treatments (**Figure 2**).

In the *acdc1*, HCNp decreased more rapidly than both other lines when well watered, consistent with earlier generations of the mutant (Blomstedt et al., 2012; Blomstedt et al., 2018). Water limitation appeared to override this developmental regulation with no significant difference in HCNp between *acdc1* and wild-type plants when water was limited. The difference in HCNp between well-watered and water-limited plants was therefore greatest in the *acdc1*, corresponding to either a slower decrease in endogenous remobilization of dhurrin, or heightened induction of dhurrin synthesis when water availability was reduced in comparison to wild-type plants. Production of dhurrin under the level of water limitation imposed may indicate that this is a direct stress response. However, as no plants maintained low-cyanogenic potential under water limitation, it was not possible to directly determine whether dhurrin plays a role in the mitigation of drought stress.

The higher HCNp across all lines when water was limited may be due to increased *de novo* biosynthesis of dhurrin, decreased remobilization, or a concentration effect due to reduced plant growth. Total dhurrin content also increased above previous levels in the 100% H_2_O treatment (**Supplementary Figure 1**), and although this did not correspond to higher HCNp due to a greater increase in the biomass of these plants, it does document on-going *de novo* synthesis. The total dhurrin content in the roots and shoots of plants when water was limited was not higher than the 100% H_2_O treatment at any harvest, and the higher concentrations observed were therefore attributable to a reduced total biomass. O’Donnell et al. (2013) found that hydroponically grown sorghum exposed to 20% PEG contained the same amount of dhurrin on a whole plant basis as non-stressed plants, also attributing the HCNp increase at least partially to a concentration effect.

Very few studies report the effect of experimental treatments on root HCNp in sorghum, in part due to the misconception that sorghum roots are not cyanogenic. This may stem from a misinterpretation of the study by Akazawa et al. (1960) where no free HCN was found in the seed or root of sorghum, a finding that was later misconstrued as signifying an absence of dhurrin in these tissues. Here, we showed clearly that sorghum roots are cyanogenic. Hydrogen cyanide potential of the root tissue was generally lower when water was limited, consistent with results for osmotic stress reported by O’Donnell et al. (2013) and in contrast to the increase observed in the shoots. There was also a significant increase in both root HCNp and total root dhurrin content in plants harvested at 35 dpg compared with 27 dpg for all treatments (**Figures 2G–I**, **Supplementary Figure 1**). This suggests that either dhurrin had been synthesized in the root tissues or transported from the shoots to the roots.

Evidently the regulatory mechanisms governing the deployment of dhurrin in the roots are different to those occurring in the shoots. The cyanogenic status of roots differs between cyanogenic species, for example, roots of eucalypts and white clover have been reported to lack cyanogenic glucosides (Hughes, 1991; Gleadow and Woodrow, 2002a), while sorghum roots and cassava tubers contain them (Jørgensen et al., 2005; O’Donnell et al., 2013). In cassava, the cyanogenic glucosides linamarin and lotaustralin are synthesized in the leaf tissue and transported to the tuber (Jørgensen et al., 2005). In sorghum, it is not confirmed whether the roots synthesize dhurrin *de novo*, or whether transport between tissues occurs. Busk and Møller (2002) found that in 5-week-old sorghum plants, the rate-limiting dhurrin biosynthetic enzyme CYP79A1 was only active in the stem, with no activity present in the leaves, leaf sheaths, or roots. From this it was deduced that in sorghum dhurrin is transported from the stem to the leaves. Selmar et al. (1996) found further evidence that dhurrin transportation may occur with the diglucoside dhurrin-6’-glucoside present in leaf guttation droplets. Diglucosides can be stably transported within plants, as seen in rubber trees (*Hevea brasiliensis*), which convert the cyanogenic monoglucoside linamarin to the diglucoside linustatin for transport from the endosperm to the seedling (Selmar et al., 1988).

In this study, the lower HCNp present in the roots, compared to the higher dhurrin concentration seen in the shoot tissue under drought, may be due to less dhurrin transported to the roots and more to the leaves and sheaths during these periods, rather than dhurrin synthesis in each individual tissue changing in response to water limitation. As few studies have analyzed the HCNp of root tissues in older plants, this is an area that would benefit from further investigation both in sorghum and in cyanogenic plants with edible underground storage organs such as cassava and taro.

### Decreases in Plant Dhurrin may Result in Higher Nitrate Concentrations

Overall, *acdc1* had higher concentrations of nitrate at the final harvest (**Figure 3**). This was particularly pronounced in the roots, which had more than double the concentration of nitrate compared to the wild-type plants. This supports the hypothesis that when less nitrogen is allocated to dhurrin, there will be a resultant increase in stored nitrate. Previous studies have found conflicting results. In osmotically stressed sorghum, high dhurrin concentrations also correlated with lower nitrate concentrations (O’Donnell et al., 2013). Conversely, Gleadow et al. (2016a) and Neilson et al. (2015) found that water limitation increased both dhurrin and nitrate concentrations in sorghum shoot tissue (root tissue was not analyzed in these studies).

Nitrate concentrations are dependent on the rate of nitrate uptake from the soil and nitrate reduction via nitrate reductase. Though the activation state of nitrate reductase does not usually change in response to variations in nitrate supply (Kaiser and Huber, 2001; Diouf et al., 2004), drought stress is associated with a decrease in nitrate reductase activity, with long-term drought leading to the inactivation and degradation of the enzyme (Foyer et al., 1998; Kaiser and Huber, 2001; Fresneau et al., 2007). Reallocation of nitrates to root tissues is found to occur under osmotic stress (Smirnoff and Stewart, 1985; Chen et al., 2012). This was observed in both the *acdc1* and wild-type plants in this study, with root nitrate concentration increasing when water was limited (**Figure 3**). Nitrate retention in roots could also be due to the reduction of nitrate transporters, with root nitrate retention in turn acting as a stress signal and activating osmotic stress related genes as thought to occur in *Arabidopsis* (Chen et al., 2012).

In this study, total nitrogen was also higher, on average, in the *acdc1* sheath and root for all treatments compared to the wild type and Sibs (**Figure 3H–I**). Though the higher nitrate concentration seen in the *acdc1* may account for the higher total N found in the roots, the *acdc1* sheaths at 15% H_2_O and 30% H_2_O did not have higher nitrate concentrations than other lines yet still had significantly greater amounts of N. The proportion of N allocated to dhurrin was lower in the *acdc1* leaf tissue at 100% H_2_O but increased under water limitation where it was equivalent to the other lines (**Figures 4A–C**). This lower allocation of N to dhurrin in the *acdc1* leaf equated to a higher proportion of N allocated to nitrate compared to the wild-type line (**Figure 4**). In agreement with this, the sorghum EMS-mutant *totally cyanide deficient 1*, which does not produce dhurrin at any stage of development, has been found to allocate more nitrogen to nitrate in the leaf tissue than wild-type plants at later stages of development (Blomstedt et al., 2018). It is difficult to state whether there is a direct trade-off between dhurrin and nitrate occurring in sorghum, particularly as the differences are tissue dependent. Here, the results are further confounded by the *acdc1* having significantly higher levels of nitrogen in all tissues for all treatments.

## Conclusions

In this study, plant age and water limitation were found to be the most important determinants of dhurrin concentration in sorghum. The *acdc1* had lower dhurrin concentrations in the leaf tissue under fully-watered conditions, though this difference was not seen when water was limited. Despite HCNp decreasing as the plants matured when water was replete, synthesis of dhurrin continued to occur with total plant dhurrin content increasing until the final harvest.

The driving factor of nitrate concentrations were genotypic differences, with the *acdc1* storing higher concentrations of nitrates in the leaves and roots than wild-type plants for all treatments. Nitrate concentrations were affected by the level of water limitation, though less so than dhurrin, where nitrates showed an opposite trend: decreasing in leaf tissues as water availability decreased, while increasing in the root tissues. Trade-offs between nitrate and dhurrin may occur with lower dhurrin concentration in the *acdc1* leaf tissue, corresponding to higher nitrate concentration compared to the wild-type line at 100% H_2_O. Growth indices in the *acdc1* were not affected by differences in dhurrin or nitrate concentrations in comparison to wild-type plants either under water-limited conditions or when fully-watered.

This study has demonstrated that dhurrin and nitrate concentrations in sorghum are highly dynamic, with regulation differing between above and below ground tissues. Changes in cyanogenic glucoside concentrations, both developmentally and in response to environmental factors, need to be considered with respect to their effect on stored nitrates for all tissues, as influencing concentrations in one tissue may affect another, particularly if transport of cyanogenic glucosides and nitrate is occurring between tissues.

## Data Availability Statement

All datasets generated for this study are included in the article/**Supplementary Material**.

## Author Contributions

Experimental studies and analyses were carried out by VR. CB, BLM, TG, and RG contributed to the design and coordination of the studies, as well as data interpretation. VR, CB, and RG drafted the manuscript. All authors contributed to the approval of the final manuscript.

## Funding

The project was supported by Australian Research Council grants LP100100434 and DP130101049. Viviana Rosati is supported by an Australian Government Research Training Program Scholarship, an AW Howard Memorial Trust Inc. Research Fellowship, and a Monash University Postgraduate Publication Award. We acknowledge the use of the facilities, and scientific and technical assistance of the Australian Plant Phenomics Facility, which is supported by the Australian Government’s National Collaborative Research Infrastructure Strategy (NCRIS).

## Conflict of Interest

The authors declare that the research was conducted in the absence of any commercial or financial relationships that could be construed as a potential conflict of interest.

## Abbreviations

*acdc1*, *adult cyanide deficient class 1*; dpg, days post germination; EMS, ethyl methanesulfonate; HCNp, hydrogen cyanide potential; RGR, relative growth rate; Sibs, sibling line; WT, wild type.
