# Supplementary material for: The Interplay Between Water Limitation, Dhurrin, and Nitrate in the Low-Cyanogenic Sorghum Mutant adult cyanide deficient class 1
Source: Front Plant Sci. 2019 Nov 15;10:1458. doi: 10.3389/fpls.2019.01458 (PMC6874135; doi:10.3389/fpls.2019.01458)
Supplement: Supplementary file 1 [file Table_1.docx]

Supplementary Material

The interplay between water limitation, dhurrin, and nitrate in the low-cyanogenic sorghum mutant adult cyanide deficient class 1

Rosati, VC, Blomstedt, CK, Møller, BL, Garnett, T and Gleadow, R 2019

**Supplementary Table 1:** Growth parameters of three *Sorghum bicolor* lines grown at 15%, 30%, and 100% field capacity of water and harvested at **(A)** 11dpg - baseline harvest before treatments commenced; **(B)** 19dpg; and; **(C)** 27dpg. WT: wild type; Sibs: siblings; *acdc1*: *adult cyanide deficient class 1* mutants. Values denote mean ± 1SE (*n*=3). Means with different letters are significantly different at P<0.05 analysed using ANOVA and Tukey’s test. Abbreviations: DM - Dry mass; LAR - leaf area ratio; NAR - net assimilation rate; R:S ratio - root:shoot ratio; SLA - specific leaf area; SLN - specific leaf nitrogen; RGR - relative growth rate. L=Line; T=Treatment; **P*<0.05; ***P*<0.01; ****P*<0.001; ns - not significant.

**(A) 11dpg**

|  | **WT** | **Sibs** | ***acdc1*** | **ANOVA** |
| --- | --- | --- | --- | --- |
| Leaf Area (cm^2^) | 4.6 (0.4) | 4.7 (0.4) | 4.9 (0.5) | ns |
| Shoot DM (g) | 0.018 (0.002) | 0.016 (0.001) | 0.016 (0.002) | ns |
| Root DM (g) | 0.017 (0.003) | 0.017 (0.002) | 0.015 (0.002) | ns |
| Biomass Total (g) | 0.035 (0.003) | 0.033 (0.003) | 0.031 (0.004) | ns |
| R:S | 1.0 (0.2) | 1.1 (0.1) | 1.0 (0.1) | ns |
| LAR (cm^2^ g^-1^) | 130 (20) | 130 (10) | 160 (10) | ns |
| SLA (cm^2^ g^–1^) | 250 (10) | 270 (5) | 310 (10) | *** |

**(B) 19dpg**

|  | **WT** | | | **Sibs** | | | ***acdc1*** | | | **ANOVA** | | |
| --- | --- | --- | --- | --- | --- | --- | --- | --- | --- | --- | --- | --- |
|  | **15% H_2_O** | **30% H_2_O** | **100% H_2_O** | **15% H_2_O** | **30% H_2_O** | **100% H_2_O** | **15% H_2_O** | **30% H_2_O** | **100% H_2_O** | **L** | **T** | **LxT** |
| Leaf Area (cm^2^) | 11 (2) | 15 (2) | 38 (3) | 20 (4) | 10 (2) | 29 (3) | 17 (3) | 22 (4) | 40 (5) | ns | * | ns |
| Height (cm) | 4.5 (0.4) | 4.9 (0.3) | 8.8 (0.3) | 4.3 (0.7) | 3.4 (0.6) | 5.9 (0.2) | 4.6 (0.5) | 4.9 (0.5) | 7.5 (0.6) | ns | ** | ns |
| Leaf DM (g) | 0.036 (0.003) | 0.042 (0.005) | 0.080 (0.009) | 0.043 (0.009) | 0.028 (0.003) | 0.060 (0.008) | 0.042 (0.010) | 0.058 (0.007) | 0.087 (0.018) | ns | * | ns |
| Sheath DM (g) | 0.021 (0.003) | 0.025 (0.003) | 0.044 (0.004) | 0.022 (0.004) | 0.023 (0.006) | 0.028 (0.003) | 0.029 (0.004) | 0.031 (0.006) | 0.043 (0.008) | ns | * | ns |
| Root DM (g) | 0.047 (0.015) | 0.054 (0.012) | 0.049 (0.005) | 0.059 (0.008) | 0.060 (0.011) | 0.066 (0.013) | 0.068 (0.010) | 0.086 (0.019) | 0.113 (0.027) | * | ns | ns |
| Biomass Total (g) | 0.10 (0.02) | 0.12 (0.02) | 0.17 (0.01) | 0.12 (0.02) | 0.11 (0.02) | 0.15 (0.02) | 0.14 (0.02) | 0.17 (0.02) | 0.24 (0.05) | * | * | ns |
| R:S | 0.8 (0.2) | 0.8 (0.1) | 0.4 (0.1) | 1.0 (0.2) | 1.2 (0.2) | 0.7 (0.1) | 1.1 (0.2) | 1.2 (0.4) | 0.9 (0.1) | ns | ns | ns |
| LAR (m^2^ g^-1^) | 110 (20) | 130 (10) | 220 (10) | 140 (18) | 90 (10) | 200 (30) | 120 (10) | 130 (30) | 180 (20) | ns | * | ns |
| SLA (m^2^ g^-1^) | 300 (50) | 370 (20) | 490 (40) | 430 (40) | 360 (60) | 500 (50) | 440 (50) | 360 (30) | 510 (50) | ns | ns | ns |
| NAR (g m^-2^ day^-1^) | 0.001 (0.001) | 0.002 (0.001) | 0.005  (0.0004) | 0.002 (0.001) | 0.001 (0.001) | 0.004 (0.001) | 0.002 (0.001) | 0.003 (0.001) | 0.008 (0.002) | ns | * | ns |
| RGR (g g^-1^ day^-1^) | 0.13 (0.03) | 0.15 (0.03) | 0.22 (0.01) | 0.16 (0.02) | 0.14 (0.02) | 0.19 (0.03) | 0.10 (0.06) | 0.17 (0.02) | 0.25 (0.03) | * | ns | ns |

**(C) 27dpg**

|  | **WT** | | | **Sibs** | | | ***acdc1*** | | | **ANOVA** | | |
| --- | --- | --- | --- | --- | --- | --- | --- | --- | --- | --- | --- | --- |
|  | **15% H_2_O** | **30% H_2_O** | **100% H_2_O** | **15% H_2_O** | **30% H_2_O** | **100% H_2_O** | **15% H_2_O** | **30% H_2_O** | **100% H_2_O** | **L** | **T** | **LxT** |
| Leaf Area (cm^2^) | 62 (9) | 75 (8) | 275 (32) | 45 (12) | 38 (11) | 273 (36) | 70 (14) | 81 (15) | 336 (54) | ns | ** | ns |
| Height (cm) | 8.3 (0.2) | 9.6 (1.9) | 16.8 (0.3) | 5.3 (0.8) | 4.7 (0.7) | 15.1 (0.7) | 6.7 (0.7) | 6.8 (0.7) | 16.0 (0.6) | ns | ** | ns |
| Leaf DM (g) | 0.16 (0.02) | 0.17 (0.02) | 0.63 (0.11) | 0.11 (0.03) | 0.13 (0.04) | 0.69 (0.11) | 0.17 (0.03) | 0.21  (0.04) | 0.78 (0.23) | ns | ** | ns |
| Sheath DM (g) | 0.09 (0.01) | 0.09 (0.01) | 0.32 (0.04) | 0.06 (0.02) | 0.05 (0.01) | 0.33 (0.06) | 0.09 (0.02) | 0.10  (0.02) | 0.45 (0.10) | ns | ** | ns |
| Root DM (g) | 0.65 (0.08) | 0.54 (0.19) | 1.06 (0.18) | 0.85 (0.30) | 0.70 (0.28) | 1.71 (0.34) | 0.62 (0.34) | 0.31  (0.09) | 1.97 (0.49) | ns | * | ns |
| Biomass Total (g) | 0.91 (0.09) | 0.80 (0.21) | 2.00 (0.26) | 1.02 (0.34) | 0.88 (0.32) | 2.73 (0.44) | 0.88 (0.34) | 0.62  (0.14) | 3.20 (0.69) | ns | ** | ns |
| R:S | 3.0 (0.5) | 2.0 (0.6) | 1.2 (0.2) | 4.4 (1.0) | 3.7 (1.0) | 1.8 (0.3) | 2.8 (1.3) | 0.9 (0.1) | 2.0 (0.5) | ns | ns | ns |
| LAR (m^2^ g^-1^) | 70 (10) | 120 (30) | 140 (10) | 80 (30) | 60 (10) | 110 (10) | 130 (40) | 140 (10) | 120 (20) | ns | ns | ns |
| SLA (m^2^ g^-1^) | 380 (10) | 440 (10) | 460 (30) | 390 (30) | 320 (30) | 410 (20) | 380 (30) | 380 (10) | 390 (50) | ns | ns | ns |
| NAR (g m^-2^ day^-1^) | 0.02 (0.001) | 0.02 (0.01) | 0.06 (0.01) | 0.03 (0.02) | 0.03 (0.01) | 0.1  (0.02) | 0.02 (0.01) | 0.01 (0.004) | 0.1  (0.03) | ns | ** | ns |
| RGR (g g^-1^ day^-1^) | 0.27 (0.03) | 0.22 (0.03) | 0.30 (0.02) | 0.21 (0.07) | 0.22 (0.05) | 0.36 (0.04) | 0.14 (0.08) | 0.15  (0.03) | 0.32 (0.03) | ns | ns | ns |

**Supplementary Table 2**: Hydrogen cyanide potential (HCNp - mg HCN g^-1^ dry mass) of three *Sorghum bicolor* lines grown at 15%, 30%, and 100% field capacity of water and harvested at 11dpg, 19dpg, 27dpg, and 35dpg for **A)** Leaf; **B)** Sheath; and **C)** Root tissues. Values denote mean ± 1SE (*n*=3). Significance at P<0.05 analyzed is shown using ANOVA and Tukey’s test. Abbreviations: WT - wild type; Sibs - Siblings; *acdc1* - adult cyanide deficient mutants; L - Line; T - Treatment; **P*<0.05; ***P*<0.01; ****P*<0.001; ns - not significant.

**A) Leaf**

|  | **WT** | | | **Sibs** | | | ***acdc1*** | | | **ANOVA** | | |
| --- | --- | --- | --- | --- | --- | --- | --- | --- | --- | --- | --- | --- |
|  | 15% H_2_O | 30% H_2_O | 100% H_2_O | 15% H_2_O | 30% H_2_O | 100% H_2_O | 15% H_2_O | 30% H_2_O | 100% H_2_O | L | T | LxT |
| 11dpg |  |  | 5.90 (0.34) |  |  | 5.40 (0.37) |  |  | 5.90 (0.35) | ns |  |  |
| 19dpg | 3.21 (0.60) | 2.21 (0.78) | 1.72 (0.44) | 2.59 (0.37) | 3.12 (0.82) | 1.73 (0.37) | 2.33 (0.46) | 2.48 (0.71) | 2.84 (0.74) | ns | ns | ns |
| 27dpg | 1.00 (0.17) | 1.14 (0.37) | 0.78 (0.27) | 1.27 (0.44) | 1.20 (0.09) | 0.67 (0.06) | 0.96 (0.19) | 1.19 (0.25) | 0.29 (0.05) | * | * | ns |
| 35dpg | 2.11^a^ (0.17) | 1.51^a^ (0.37) | 0.65^b^ (0.06) | 2.50^a^ (0.37) | 2.52^a^ (0.57) | 0.60^b^ (0.03) | 2.68^a^ (0.59) | 1.73^a^ (0.36) | 0.28^c^ (0.02) | * | ** | * |

**B) Sheath**

|  | **WT** | | | **Sibs** | | | ***acdc1*** | | | **ANOVA** | | |
| --- | --- | --- | --- | --- | --- | --- | --- | --- | --- | --- | --- | --- |
|  | 15% H_2_O | 30% H_2_O | 100% H_2_O | 15% H_2_O | 30% H_2_O | 100% H_2_O | 15% H_2_O | 30% H_2_O | 100% H_2_O | L | T | LxT |
| 11dpg |  |  | 5.90 (0.34) |  |  | 5.40 (0.37) |  |  | 5.90 (0.35) | ns |  |  |
| 19dpg | 2.42 (0.44) | 2.60 (0.42) | 1.60 (0.14) | 2.50 (0.42) | 2.81 (0.66) | 1.89 (0.25) | 2.35 (0.54) | 2.30 (0.25) | 1.82 (0.18) | ns | * | ns |
| 27dpg | 0.65 (0.09) | 0.69 (0.10) | 0.36 (0.04) | 0.68 (0.12) | 0.81 (0.19) | 0.35 (0.05) | 0.93 (0.10) | 1.04 (0.11) | 0.30 (0.03) | ns | ** | ns |
| 35dpg | 1.36^a^ (0.29) | 1.03^a^ (0.25) | 0.40^b^ (0.10) | 1.27^a^ (0.12) | 1.04^a^ (0.09) | 0.46^b^ (0.08) | 1.86^a^ (0.28) | 1.39^a^ (0.28) | 0.20^c^ (0.10) | ns | ** | * |

**C) Root**

|  | **WT** | | | **Sibs** | | | ***acdc1*** | | | **ANOVA** | | |
| --- | --- | --- | --- | --- | --- | --- | --- | --- | --- | --- | --- | --- |
|  | 15% H_2_O | 30% H_2_O | 100% H_2_O | 15% H_2_O | 30% H_2_O | 100% H_2_O | 15% H_2_O | 30% H_2_O | 100% H_2_O | L | T | LxT |
| 11dpg |  |  | 0.46 (0.14) |  |  | 0.45 (0.05) |  |  | 0.36 (0.06) | ns |  |  |
| 19dpg | 0.16 (0.02) | 0.30 (0.08) | 0.53 (0.08) | 0.19 (0.04) | 0.18 (0.04) | 0.53 (0.15) | 0.14 (0.03) | 0.29 (0.09) | 0.35 (0.07) | ns | * | ns |
| 27dpg | 0.01 (0.003) | 0.06 (0.02) | 0.12 (0.02) | 0.02 (0.01) | 0.05 (0.02) | 0.14 (0.03) | 0.11 (0.04) | 0.25 (0.05) | 0.24 (0.07) | * | * | ns |
| 35dpg | 0.17 (0.05) | 0.39 (0.10) | 0.32 (0.04) | 0.11 (0.03) | 0.24 (0.04) | 0.40 (0.04) | 0.25 (0.05) | 0.53 (0.05) | 0.48 (0.05) | * | ** | ns |


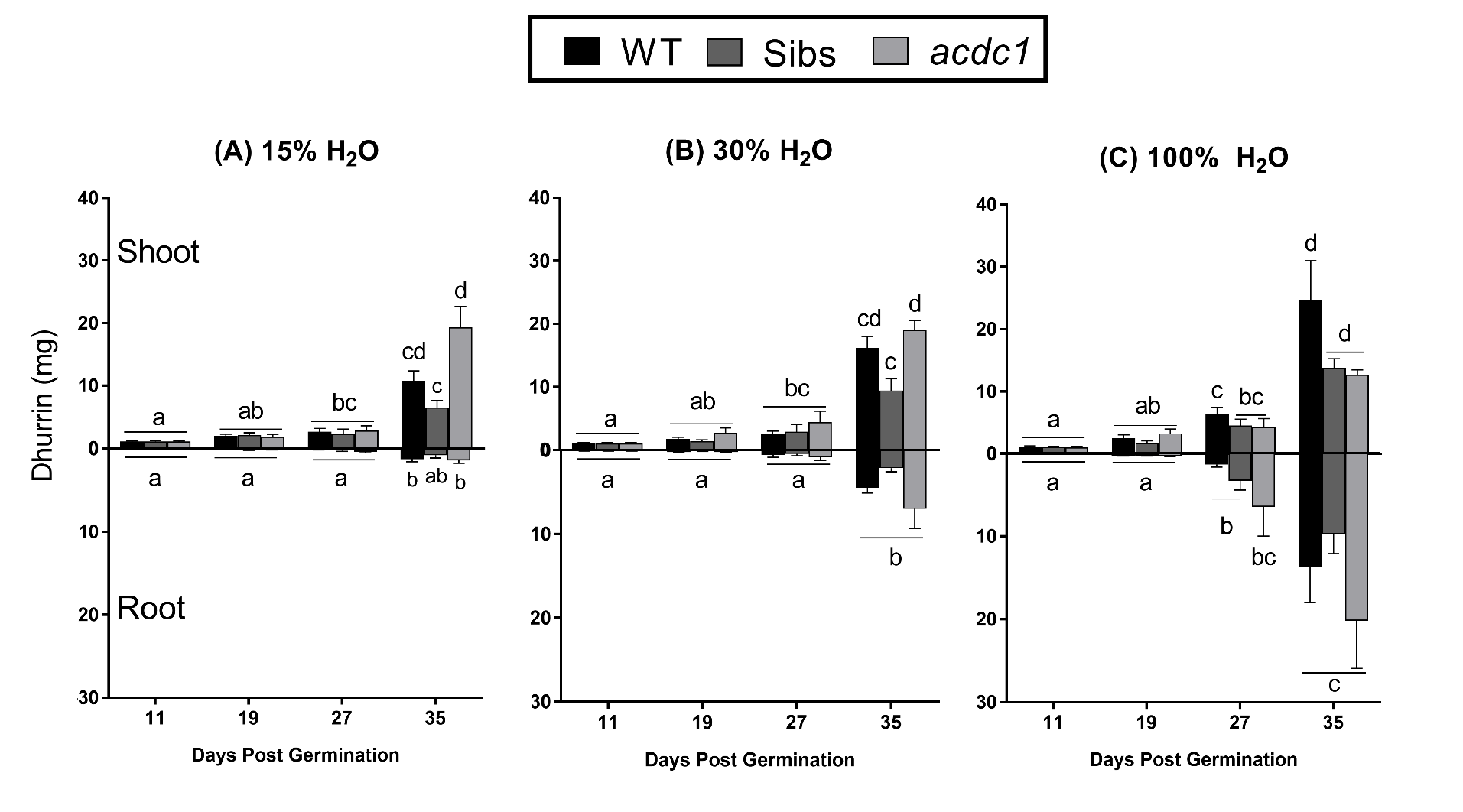


**Supplementary Figure 1:** Total dhurrin content (mg) of shoot and root tissues of WT - wild type; Sibs - siblings; and *acdc1* - *adult cyanide deficient class 1* sorghum lines grown at **(A)** 15% H_2_O; **(B)** 30% H_2_O; and (C) 100% H_2_O. Values denote mean ± 1SE (*n*=3), means with different letters are significantly different at P<0.05, analyzed using ANOVA and Tukey’s test.
